# The fate of anti-HLA antibodies following liver transplantation

**DOI:** 10.3389/fneph.2024.1403096

**Published:** 2024-06-12

**Authors:** Douglas J. Norman, C. Kristian Enestvedt, Willscott E. Naugler, Rouella Erhan, Carley A. Shaut

**Affiliations:** ^1^ Laboratory of Immunogenetics and Transplantation, Oregon Health & Science University, Portland, OR, United States; ^2^ Section of Transplantation Medicine, Division of Nephrology, Department of Medicine, Oregon Health & Science University, Portland, OR, United States; ^3^ Division of Abdominal Organ Transplantation, Department of Surgery, Oregon Health & Science University, Portland, OR, United States; ^4^ Division of Gastroenterology and Hepatology, Department of Medicine, Oregon Health & Science University, Portland, OR, United States

**Keywords:** liver transplant, anti HLA antibodies, donor specific antibodies, HLA class I and class II typing, liver graft survival

## Abstract

**Introduction:**

Liver transplant recipients may have pre-formed anti-HLA antibodies directed to mismatched HLA of the liver donor (donor specific antibodies, DSA) or not directed to the liver donor (non-donor specific, non-DSA). We observed the fate of these antibodies (DSA and non-DSA) at 12 months after transplant.

**Methods:**

Patients transplanted between 4/2015 and 12/2018 (N = 216) who had anti-HLA antibody measurements at both transplant and 12 months posttransplant (N = 124) and with DSAs at transplant (N = 31) were considered informative for a paired analysis of the natural history of DSA and non-DSA following liver transplantation.

**Results:**

Class I DSAs and non-DSAs decreased between transplant and 12 months; however, Class I DSAs essentially disappeared by 12 months while Class I non-DSAs did not. Anti-HLA Class II DSAs performed differently. While there was a significant drop in values between transplant and 12 months, these antibodies mostly persisted at a low level.

**Discussion:**

Our study demonstrated a significant difference in the kinetics of DSA compared to non-DSA following liver transplantation, most profoundly for anti-HLA Class I antibodies. Class I DSAs were mostly absent at 12 months while Class II DSAs persisted, although at lower levels. The mechanisms of reduction in anti-HLA antibodies following liver transplantation are not completely understood and were not pursued as a part of this study. This detailed analysis of Class I and Class II DSAs and non-DSAs represents and important study to explore the change in antibodies at one year from liver transplantation.

## Introduction

Patients undergoing liver transplantation may have developed circulating anti-HLA antibodies due to previous blood transfusions, a prior transplant, pregnancy or a combination of these. These preformed anti-HLA antibodies are either against mismatched HLA antigens of the liver donor (donor specific antibodies, DSA) or against other HLA antigens (non-donor specific, non-DSA). In general, donor specific anti-HLA antibodies have no impact on posttransplant outcomes (1–10). Exceptions may occur in second transplants and in patients with a high level of anti-HLA Class II (DP, DQ and DR) antibodies (11–32). Anti-HLA antibodies decrease in amount after liver transplantation (33–36) and Class I DSAs generally disappear (33). In contrast, Class II DSAs are more likely to persist following liver transplantation (35). The absence of consequences of DSA in liver transplantation as compared to kidney or heart transplantation is not fully understood. Frequently cited theories for this phenomenon include a very large capillary bed (100x greater in the liver than in kidneys) expressing HLA Class I antigens (37–39), and secretion of soluble HLA Class I and Class II antigens capable of binding donor specific antibodies (38).

The primary focus of previous studies of anti-HLA antibodies in liver transplant recipients has centered on the potential harm caused by DSAs. This was not the focus of our study although data are presented that confirm previous findings on graft survival. Rather, the primary aim of this study was to observe the fate of all anti-HLA antibodies following liver transplantation. We aimed to determine if there are different patterns of clearance of DSAs compared with non-DSAs and if these patterns suggest mechanisms of anti-HLA antibody clearance. To our knowledge, published accounts of a comparison between non-DSA and DSA in liver transplant recipients are absent in the literature. We assessed the fate, at 12 months after transplant, of anti-HLA antibodies present at transplant, assessing both donor specific and non-donor specific antibodies.

## Methods

### Patients

All patients who received a liver transplant at OHSU between April 2015 and December 2018 (N = 216) were considered in this IRB-approved retrospective study. Among those patients, only patients who had anti-HLA antibody measurements at the time of transplant and at 12 months posttransplant were considered informative (N = 124). Thirty-five patients had no testing, 49 patients had no testing at transplant and eight patients had testing at transplant but no testing at 12 months after transplant. Among the 124 patients, we studied patients with DSAs at transplant to fulfill our primary aim for a paired analysis of the natural history of DSAs and non-DSAs. For these thirty-one patients, some of whom had more than one DSA, we studied all DSAs present at transplant. For comparison, we studied up to five non-DSAs for each patient with a DSA. **Table 1** lists the MFIs of the 39 Class I DSAs and 37 Class II DSAs at the time of transplant and at 12 months after transplant.

**Table 1:** MFI of the 39 Class I DSAs and 37 Class II DSAs at transplant and 12 months after transplant.

Donor Specific Antibodies					
Class I DSA	MFI at Transplant	MFI at 12 Months	Class II DSA	MFI at Transplant	MFI at 12 Months
A1	1323	25	DP4	12416	403
A2	25524	35	DP6	10950	726
A2	21987	23	DP18	21960	2293
A2	18889	134	DP20	6549	252
A2	2916	78	DQ2	5224	158
A2	2867	7	DQ2	3862	1656
A3	17037	159	DQ5	10239	33
A3	4623	31	DQ6	15557	280
A3	2561	37	DQ7	19414	23553
A11	2179	1	DQ7	3979	25
A24	24837	77	DQ7	3133	1883
A24	4168	19	DQ8	5402	1643
A26	23181	1	DQ8	4628	118
A30	23832	22	DR1	20224	1343
A34	2495	6	DR4	16319	190
A68	19259	32	DR4	14932	158
A68	2867	69	DR4	3328	13
B7	21867	7	DR4	2840	317
B7	19314	15	DR4	1721	390
B7	4364	153	DR4	1335	675
B13	24362	1438	DR7	19474	586
B13	7378	74	DR7	1572	537
B27	21157	57	DR7	1556	12
B27	14727	54	DR7	1101	408
B35	23434	1081	DR8	24009	300
B35	7968	43	DR8	22609	1730
B35	1924	1	DR12	23003	523
B37	1625	177	DR13	20767	5610
B39	13228	1	DR13	5712	4731
B44	20912	15	DR14	18867	5288
B44	4944	194	DR14	1439	1301
B44	3251	37	DR52	24136	2182
B50	19731	32	DR52	4734	4809
B50	16150	1	DR52	4385	176
B53	25845	32	DR52	3589	58
B53	13890	53	DR52	2046	1625
B56	11642	661	DR53	5047	626
B58	26533	22			
B60	20675	273			

**Table 2** lists the demographics of the patients included in the study. Differences were noted between patients with and without preformed anti-HLA antibodies at the time of transplant.

**Table 2:** Demographics of the patients included in this study.

Patient Characteristics		All N = 124	DSA N = 31	HLA, No DSA N = 71	No HLA N = 22	Class I and II DSA N = 11
Age		55.3	52.3	56.5	55.7	46.7
Sex = F		54 (44%)	26 (84%)	24 (34%)	3 (14%)	11 (100%)
White Race		108 (87%)	24 (77%)	61 (86%)	21 (95%)	10 (91%)
MELD		29.0	30.7	28.1	29.4	32.0
Native Liver Disease						
Alcohol		21 (17%)	5 (16%)	10 (14%)	6 (27%)	3 (27%)
Hepatitis C		32 (26%)	6 (19%)	22 (31%)	4 (18%)	2 (18%)
Primary Liver Malignancy		34 (27%)	4 (13%)	23 (32%)	7 (32%)	0 (0%)
NASH		13 (10%)	6 (19%)	7 (10%)	0 (0%)	2 (18%)
Primary Biliary Cirrhosis		5 (4%)	5 (16%)	0 (0%)	0 (0%)	2 (18%)
Primary Sclerosing Cholangitis		7 (6%)	2 (7%)	3 (4%)	2 (9%)	0 (0%)
Other		12 (10%)	3 (10%)	6 (9%)	3 (14%)	2 (18%)
ABO	O	56 (45%	14 (45%)	36 (51%)	6 (27%)	4 (36%)
	A	45 (36%)	12 (39%)	23 (32%)	10 (46%)	6 (55%)
	B	17 (14%)	2 (6%)	9 (27%)	6 (27%)	1 (9%)
	AB	6 (5%)	3 (10%)	3 (4%)	0 (0%)	0 (0%)

### HLA typing and detection and measurement of anti-HLA antibodies

Molecular HLA typing of donors was performed at HLA-A, B, C, DRB1, DRB3,4,5, DQA1, DQB1 and DPB1 using LinkSeq ABCDRDQDP+ 384 Typing kit (One Lambda Thermo Fisher). Anti-HLA antibody detection was performed on EDTA-treated sera using Class I and II Labscreen Single antigen beads (One Lambda Thermo Fisher) on a Luminex platform. A normalized mean fluorescence intensity (MFI) of >1000 was considered positive for most antigens, while a cutoff of 2000 MFI was used for DPB1 with no common epitope reactivity patterns. MFI is a semi quantitative measure of the level of antibodies present. We considered antibodies to HLA A, B, DP, DQ and DR informative for our analysis. In identifying antibodies to study, we eliminated those considered false positive (cryptic epitope patterns, pan-DR, and common false positive beads not associated with epitopes).

### Immunosuppression

We used a triple therapy immunosuppression regimen including tacrolimus, prednisone and azathioprine. Induction was typically limited to corticosteroids and occasional patients received mycophenolate mofetil instead of azathioprine.

### Statistics

We performed several different analyses using a Wilcoxon paired, non-parametric, two-tailed technique. We analyzed outcomes for changes in MFI between transplant and 12 months for the following: All DSA, all non-DSA, Class I DSA, Class I non-DSA, Class II DSA and Class II non-DSA. Patients served as their own controls when observing changes in DSA and non-DSA MFIs from transplant to 12 months. Percent reductions in MFI, between transplant and 12 months, for both DSA and non-DSA, were calculated and used for comparisons. For those patients who possessed both anti Class I and anti-Class II DSAs at transplant, the reduction in MFI from transplant to 12 months for Class I antibodies and Class II antibodies was also compared.

## Results

Thirty-one patients had 76 DSAs identified in the single antigen bead assays performed at transplant. Twenty-two patients had Class I DSAs (N = 39), and twenty patients had Class II DSAs (N = 37). In the same single antigen bead assays performed at transplant, we identified 103 Class I non-DSAs and 78 Class II non-DSAs for comparison. The non-DSAs chosen were those with the highest MFIs, if there were more than five non-DSAs. Eleven patients had both Class I and Class II DSAs.

**Figure 1:**
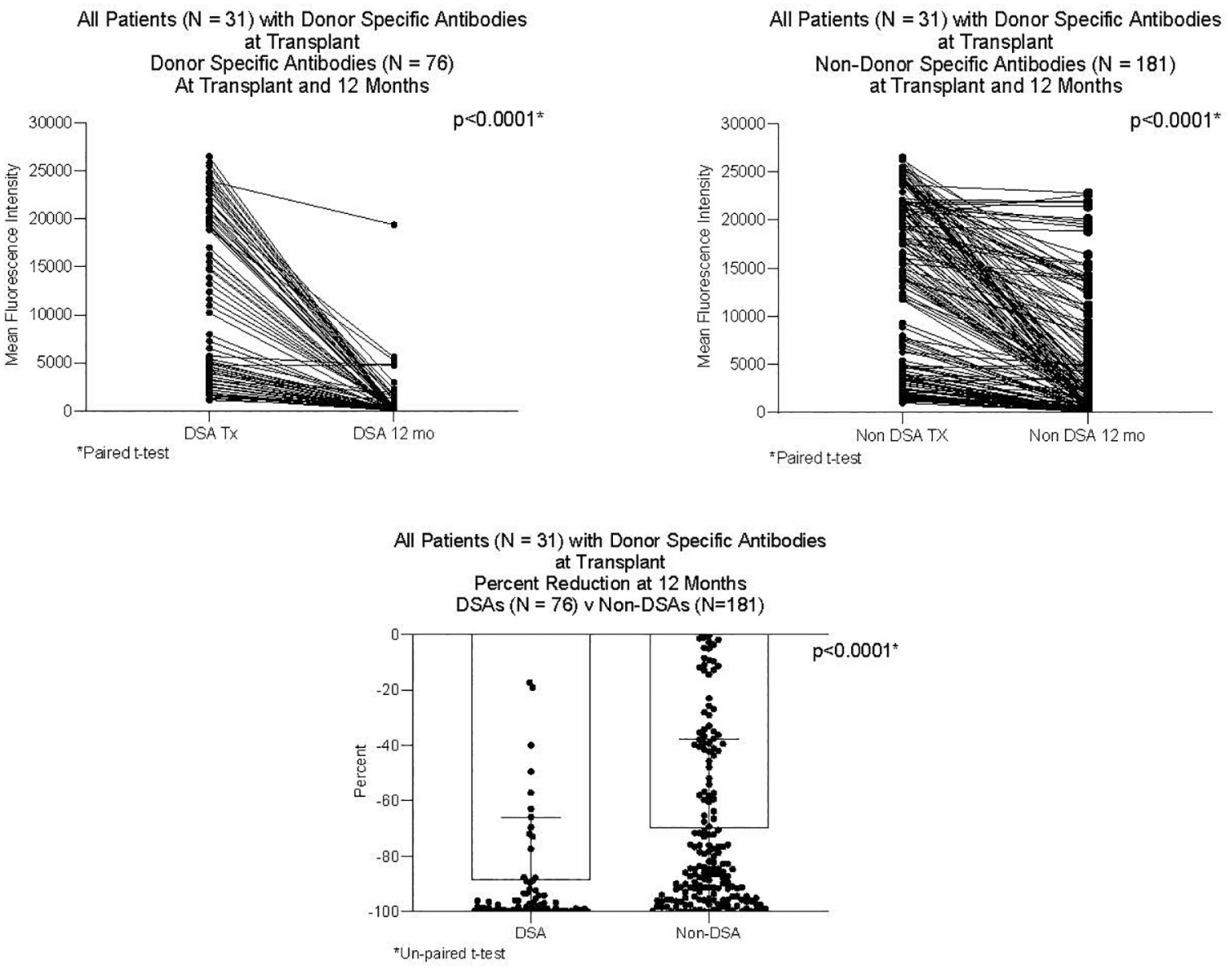
MFI of DSAs and non-DSAs at transplant and 12 months after transplant. Thirty-one patients had DSAs. The Figure compares seventy-six DSAs and 181 non-DSAs from those patients. Each line represents the value of an individual antibody at transplant and again at 12 months. There was a significant reduction in MFI by 12 months for both DSAs and non-DSAs, although the percent reduction for DSAs was significantly greater than for non-DSAs.

**Figure 2:**
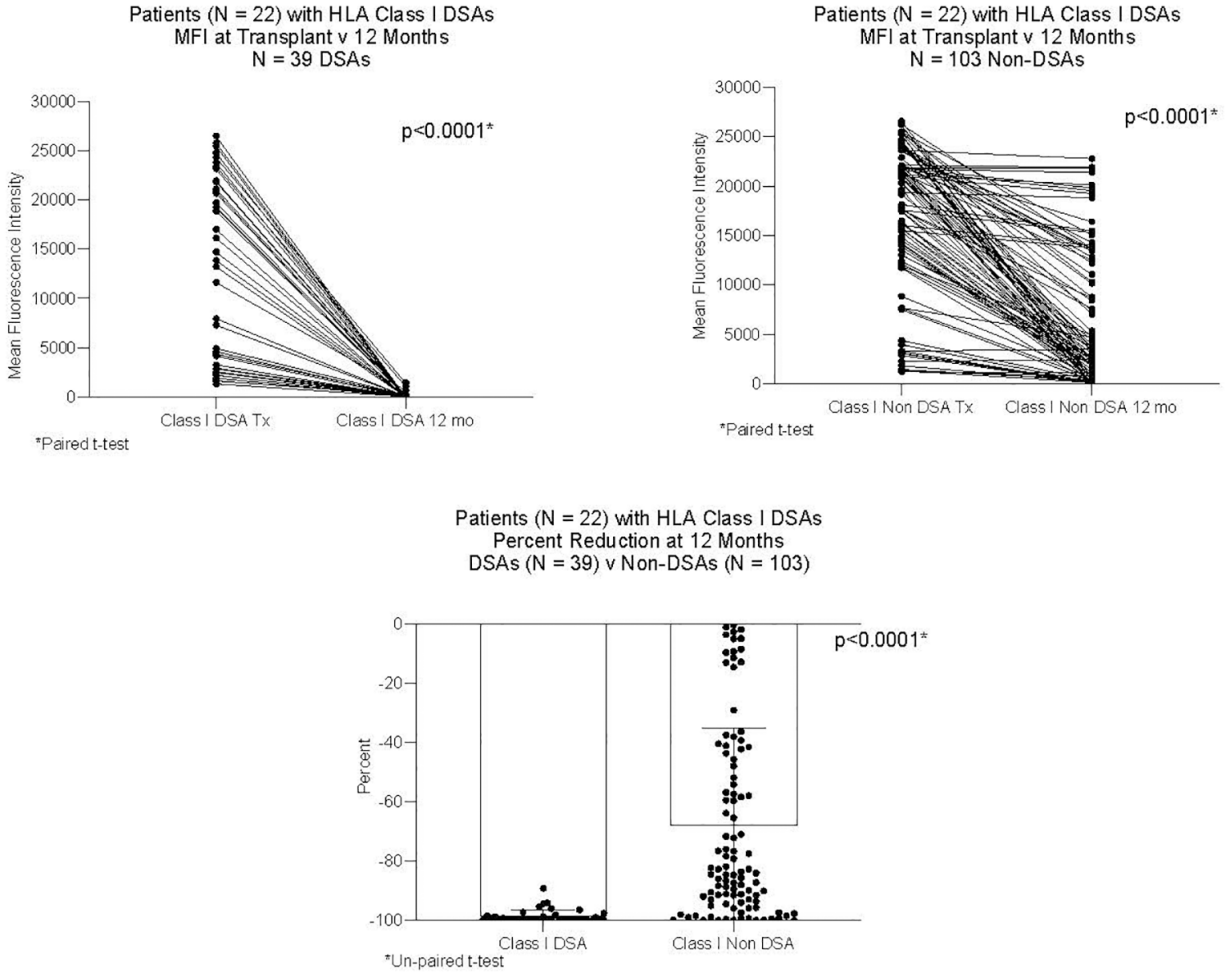
MFI of Class I DSAs and Class I non-DSAs at transplant and 12 months after transplant. Twenty-two patients had Class I DSAs. The Figure compares 39 DSAs and 103 non-DSAs from those patients. Each line represents the value of an individual antibody at transplant and again at 12 months. There was a significant reduction in MFI by 12 months for both DSAs and non-DSAs, although the percent reduction for DSAs was significantly greater than for non-DSAs.

**Figure 3:**
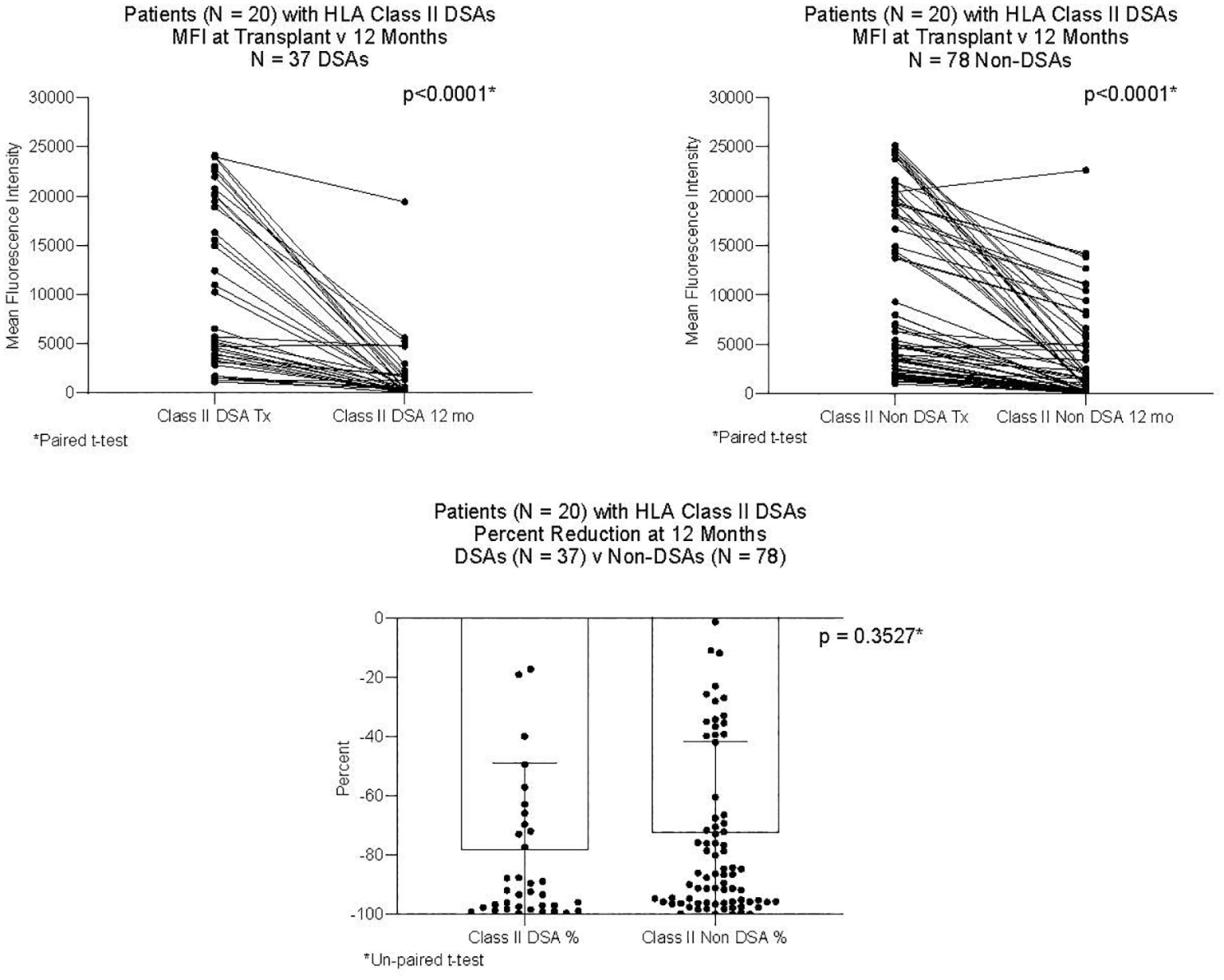
MFI of Class II DSAs and Class II non-DSAs at transplant and 12 months after transplant. Twenty patients had Class II DSAs at transplant. The Figure compares 37 DSAs and 78 non-DSAs from those patients. Each line represents the value of an individual antibody at transplant and again at 12 months. There was a significant reduction in MFI by 12 months for both DSAs and non-DSAs, although the percent reduction for DSAs was not different from that of non-DSAs.

**Figure 4:**
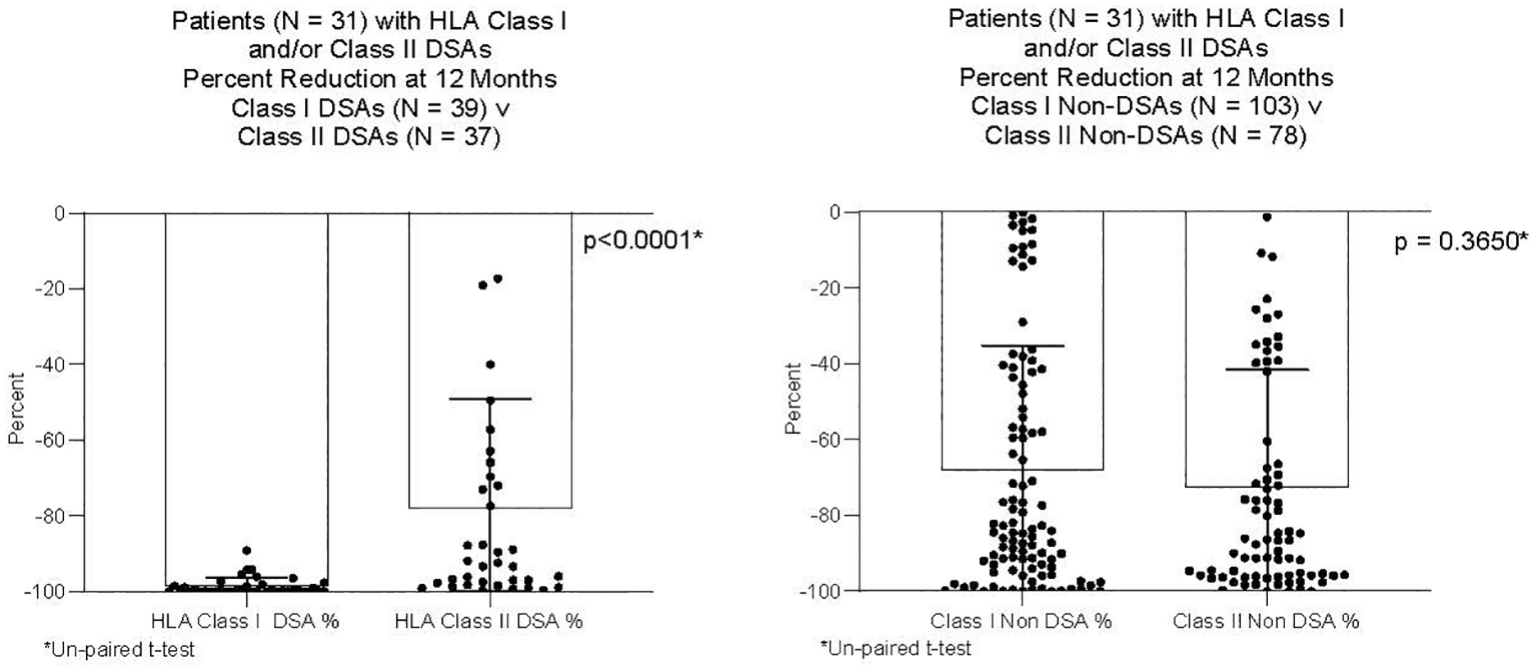
Percent reduction in MFI from transplant to 12 months after transplant. Thirty-one patients had Class I and/or Class II DSAs. The Figure compares 39 Class I DSAs to 37 Class II DSAs from those patients; and, 103 Class I non-DSAs to 78 Class II non-DSAs from those patients. The percent reduction was significantly greater for Class I DSAs compared to Class II DSAs. There was no difference in percent reduction between Class I non-DSAs and Class II non-DSAs.

DSAs and non-DSAs decreased significantly between transplant and 12 months. However, while DSAs mostly disappeared between transplant and 12 months, non-DSAs did not (**Figure 1**). The difference between DSA and non-DSA was most striking for Class I antibodies. The percent MFI reduction between transplant and 12 months was significantly greater for the Class I DSAs compared with the non-DSAs (**Figure 2**). Class II DSAs performed differently from Class I DSAs between transplant and 12 months. While there still was a significant drop in the MFI values between transplant and 12 months, these antibodies mostly persisted at a low level and the percent reduction of Class II DSA was not significantly different from the percent reduction of Class II non-DSA (**Figure 3**). The percent reduction for Class I DSA was significantly greater than for Class II DSA (**Figure 4**). Among the 11 patients with both Class I and II DSAs, the percent reduction in Class I was also significantly greater than for Class II.

**Figure 5:**
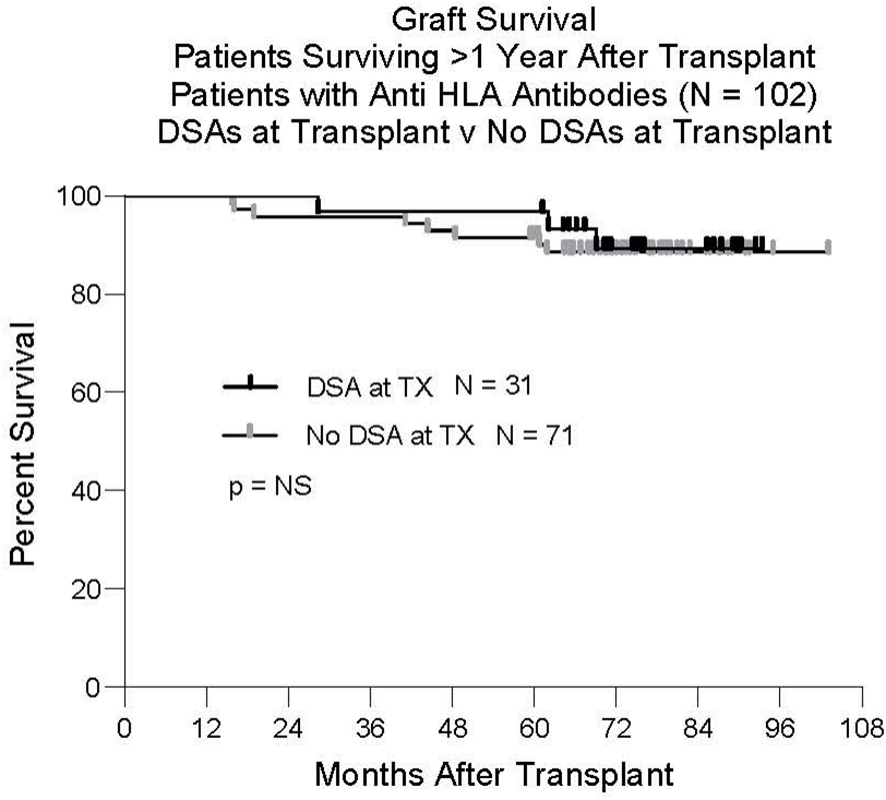
Graft survival. One-hundred-two patients had anti HLA antibodies at transplant. Thirty-one had Class I and/or Class II DSAs. Seventy-one did not have DSAs at transplant. There was no difference in outcome between these groups.

Although it was not the primary focus of this study to analyze graft survival in relation to the presence of anti-HLA DSAs, we found that graft survival of patients with DSAs at transplant was similar to that of patients without DSAs at transplant (**Figure 5**). For this analysis, we studied only patients who survived the first year. Twelve of the 136 patients tested for the presence of antibodies at transplant failed before one year. Six patients died with function and six had graft failure. Four patients did not have anti HLA antibodies. Eight patients had anti HLA antibodies but none of these had DSAs.

## Discussion

There are several overviews in the published literature documenting that DSAs tend to disappear after liver transplant (40–43). Using established DSA monitoring assays (44, 45), our study demonstrated a significant difference in the fate of DSAs compared to non-DSAs following liver transplantation. We used patients as their own controls when comparing the reduction in DSA to the reduction in non-DSA. The difference between DSA and non-DSA was most striking for anti-HLA Class I antibodies. There was also a significance difference between Class I and Class II DSAs. Class I DSA was mostly absent at 12 months while Class II DSA persisted, although at lower levels. The differential outcomes of Class I DSAs and Class II DSAs have been previously described (33) but not with the detailed comparison to non-DSAs and the large cohort present in this study.

The mechanisms of reduction in anti-HLA antibodies following liver transplantation are not completely understood and were not pursued as a part of this study. A reduction in non-DSA following liver transplantation is likely due to clearance of antibodies in concert with a natural decrease in the production of antibodies, enhanced by the use of immunosuppression. The further reduction in DSA is likely to be related to the liver allograft itself. While no mechanism has been proven, authors have speculated that the liver’s large capillary bed (21 square meters) contributes to the disappearance or reduction in DSA (37–39). HLA molecules expressed on endothelial cells in this large capillary bed could potentially bind donor specific anti-HLA antibodies. If so, this would have to occur without activating complement or propagating other inflammatory processes that could damage the liver. Another previously mentioned mechanism is the secretion of cell free HLA molecules by the liver that could bind donor specific antibodies in the circulation. The differential handling of DSA versus non-DSA also raises a possibility of tolerance induction. However, anti-Class I DSAs disappear in nearly all patients while tolerance (the ability to remove immunosuppression) occurs in only 10–15% of liver transplant recipients (46).

The reasons for the differential handling of anti-Class II antibodies compared with anti-Class I are not clear. If one implicates the binding and removal of antibodies by liver tissue as the primary mechanism, the absence of expression of Class II on most liver cells could explain the persistence of anti-Class II DSAs.

The strength of this observational study derives primarily from the large number of patients who had high titer donor specific antibodies at the time of transplant and who also had high titer non-donor specific antibodies. This study was only possible because it had been previously determined that DSAs have almost no adverse consequences in liver transplantation (confirmed by our analysis in the present study) and therefore DSAs are not assessed and avoided prior liver transplantation. Indeed, pre transplant crossmatches are only performed in liver transplantation when patients are considered for multi-visceral or other multi-organ transplants that include the liver or a retransplant is considered after graft failure from severe antibody mediated rejection. This is not true for other organs, in which donors with HLA antigens against which a recipient has antibodies are avoided because of their demonstrated adverse effects on outcomes (42).

A weakness of this study is the fact that it is retrospective and observational only. No attempt was made to determine the mechanisms of antibody reduction observed. Binding of antibodies to the endothelia of the large capillary beds of livers would require serial liver biopsies. The presence of secreted HLA molecules in liver transplant recipients and their potential for binding anti-HLA antibodies could be studied in the future. Donor-recipient interactions relative to gender, blood type, and age were also not considered for their impact on DSA and non-DSA expression. The cause of liver failure, while described as a demographic feature, was not analyzed as a predictor of *de novo* DSA or non-DSA. For instance, those patients with PSC or PBC as an indication for liver transplantation – both of which are considered on the auto-immune spectrum - may have a propensity for forming *de novo* antibodies.

## Conclusion

This was a detailed assessment of DSA and non-DSA expression against both Class I and Class II HLA that explored the change in antibodies at one year from liver transplantation. A more complete picture of this temporal change is crucial as a first step toward understanding how the effects of antibody exposure to the liver graft evolves and should provide direction for future investigations. These efforts are particularly important as the transplant community develops a more nuanced understanding of AMR in liver transplantation.

## Data availability statement

The original contributions presented in the study are included in the article/supplementary material, further inquiries can be directed to the corresponding author/s.

## Ethics statement

The studies involving humans were approved by Institution Review Board, Oregon Health & Science University. The studies were conducted in accordance with the local legislation and institutional requirements. The ethics committee/institutional review board waived the requirement of written informed consent for participation from the participants or the participants’ legal guardians/next of kin because this was a retrospective study of data collected for clinical reasons and no PHI is included.

## Author contributions

DN: Conceptualization, Data curation, Formal analysis, Investigation, Methodology, Writing – original draft, Writing – review & editing. CE: Formal analysis, Writing – review & editing. WN: Formal analysis, Writing – review & editing. RE: Data curation, Methodology, Writing – review & editing. CS: Data curation, Formal analysis, Methodology, Writing – review & editing.
